# NEIGHBORHOOD AND PSYCHOSOCIAL PREDICTORS OF ALLOSTATIC LOAD AMONG LATINX ADULTS IN THE UNITED STATES

**DOI:** 10.1093/geroni/igae098.3234

**Published:** 2024-12-31

**Authors:** Angela Gutierrez, Adil Supiyev, Courtney Thomas Tobin, Barış Sevi, Graciela Muniz Terrera

**Affiliations:** Western University of Health Sciences, Pomona, California, United States; Ohio University Heritage College of Osteopathic Medicine, Athens, Ohio, United States; University of California Los Angeles, Los Angeles, California, United States; MEF University, İstanbul, Istanbul, Turkey; Ohio University, Athens, Ohio, United States

## Abstract

Prior research highlights the independent roles of neighborhood and psychosocial risk and protective factors for accelerated physiological aging. However, the combined role of risk and protective neighborhood and psychosocial factors on allostatic load (AL) among middle-aged and older Latinx adults in the U.S. remains unclear. Utilizing data from Latinx adults ages 50 and older (n = 319) in the Health and Retirement Study (Time 1 = 2006/2008; Time 2 = 2010/2012; Time 3 = 2014/2016), this study aimed to examine the role of neighborhood (perceived physical disorder, neighborhood social cohesion) and psychosocial (loneliness, social support) factors in shaping baseline AL and change in allostatic load. Linear mixed models were used to estimate baseline AL and rate of change in AL, adjusting for sociodemographic factors. Higher loneliness was associated with higher baseline AL, p = 0.028. This association persisted when considering neighborhood factors, p = 0.025. When social support was added to the model, the association between loneliness and AL was attenuated and no longer statistically significant, p = 0.174. Findings provide evidence of a positive association between loneliness and baseline allostatic load. Finding also highlight social support as a protective factor for baseline AL among Latinx adults. Health promotion efforts should consider implementing culturally relevant approaches to foster social support and to promote healthy aging among Latinx adults.

